# Memory

**Published:** 1868-12

**Authors:** 


### MEMORY,

Like every other faculty, is cultivatable. It is improved by exercise, and like a good friend, the more we trust it the better will it serve us. But memory may be aided. For example: you may take a newspaper or a magazine, perhaps several, and it may require inconvenient attention to bear in mind when the time expires for which you have paid for each. Now we will give you a lesson in "Memory made Easy."

☞ Make all your subscriptions payable on New Year's day, and lay by the amount long before the time, so as to make assurance doubly sure, and let all be paid at once. Thus a publisher will know his position at the end of each year, and you be saved from a multitude of resolutions, with all their uncomfortable attendants; among which are a half mad, half accusatory feeling embodied in the expression, with an appropriate gesture, as likely as anything else to be a kind of gritting of the teeth—"Well, I must send on my subscription to-morrow." But it so happens, and not seldom, that the collector comes before *your* to-morrow, and you have cheated your publisher out of ten per cent. of his bill without benefiting yourself a single penny by the fraud.

No insinuations towards *our* subscribers. We have no delinquents. We do not believe in the morality of affording opportunity for such a dereliction. There is no consistency in saying daily, "Lead us not into temptation," and as habitually tempt others to get in debt by offers of credit. The "Forerunner" of this Journal is the Inevitable Dollar, and it is against public morals, and to the ruin of many a benevolent and hard-working publisher, that pre-payment is not the universal law of the press; it is the most bootless ruin in the whole commercial world, for it all falls on one man, while the pecuniary benefit to each, of a multitude, is too insignificant to be appreciable.

The less a debt is, the greater is the turpitude of not paying it. There is a looseness of feeling on this subject not easily accounted for, and more surprising still, "Religious Newspapers" suffer more heavily from this cause, than any other class.

